# Hydrophobic Shape-Memory Biocomposites from Tung-Oil-Based Bioresin and Onion-Skin-Derived Nanocellulose Networks

**DOI:** 10.3390/polym12112470

**Published:** 2020-10-25

**Authors:** Sunanda Sain, Dan Åkesson, Mikael Skrifvars, Souvik Roy

**Affiliations:** 1 Swedish Centre for Resource Recovery, University of Borås, SE-501 90 Borås, Sweden; mikael.skrifvars@hb.se; 2 Joseph Bank Laboratories, School of Chemistry, University of Lincoln, Lincoln LN6 7DL, UK; sroy@lincoln.ac.uk

**Keywords:** biocomposite, thermosetting resin, creep, thermal analysis

## Abstract

The fabrication of smart biocomposites from sustainable resources that could replace today’s petroleum-derived polymer materials is a growing field of research. Here, we report preparation of novel biocomposites using nanocellulose networks extracted from food residue (onion skin) and a vegetable oil-based bioresin. The resin was synthesized via the Diels-Alder reaction between furfuryl methacrylate and tung oil at various ratios of the components. The onion-skin-extracted cellulose nanofiber and cellulose nanocrystal networks were then impregnated with the resins yielding biocomposites that exhibited improved mechanical strength and higher storage modulus values. The properties of the resins, as well as biocomposites, were affected by the resin compositions. A 190–240-fold increase in mechanical strength was observed in the cellulose nanofiber (CNF) and cellulose nanocrystal (CNC)-reinforced biocomposites with low furfuryl methacrylate content. The biocomposites exhibited interesting shape-memory behavior with 80–96% shape recovery being observed after 7 creep cycles.

## 1. Introduction

Commercial plastic products are derived from petrochemical resources and therefore, replacing them with renewable biobased materials sourced by sustainable means fits with our current strive towards circular economy. In this context, biocomposites composed of bioresins, thermosetting resins typically derived from vegetable oils [1], and natural fibers as reinforcements would be suitable alternatives to the commercial plastics. Vegetable oils are promising raw materials for bioresin synthesis because of their triglyceride structure, together with the renewable nature, low-cost and biodegradability. Moreover, vegetable oil-based polymers offer several advantages over other bio-based polymers, including their hydrophobic nature which could complement other hydrophilic components and their tuneable properties [2]. Rapeseed, soybean, linseed, tung, castor, sunflower, and cashew nut shell liquid have all been explored for synthesis of various polymers, including polyurethane, polyesters, polyethers, and polyolefins [1,2,3,4,5,6]. However, most of these triglyceride molecules often require further functionalization before they can be polymerized. In this context, tung oil (TO) is particularly advantageous as its reactive triene groups eliminates the need for additional modification steps [7,8].

TO is primarily used in the paint and varnish industry due to its fast-oxidative drying property. The conjugated triene functionality in TO, originating from α-eleostearic acid (*cis*-9, *trans*-11, *trans*-13-octadecatrienoic acid) units, allows it to undergo various polymerization reactions, including thermal, cationic, free radical and the Diels-Alder (DA) reaction with different olefinic co-monomers [9,10,11,12,13,14,15]. Furthermore, the ratio between TO and co-monomers could be modified to tune the property of resulting polymers and expand the scope of their applications [11,12,16,17]. The long hydrocarbon chain along with olefinic functionalities of TO suggest that it can potentially be applied in shape-memory polymers [2,16,17]. Shape-memory behavior is defined as the ability of a material to retain a temporary shape upon mechanical deformation until application of an external stimulus which leads to shape-recovery [16,18]. In TO, the double bonds can provide the crosslinking points for co-monomers, regulating the permanent shape of the material, and the flexible alkyl groups can constitute the switching segment and impart ductility, enabling shape-recovery. However, examples of TO-based shape-memory polymers are scarce in literature and most of them are based on polyurethane structure [2].

We envisaged that integration of aromatic acrylate moieties into TO triglyceride structure would be a convenient way to synthesize new bioresins, as well as biopolymers that are capable of exhibiting this kind of shape-memory behavior [19]. Furfuryl methacrylate (FMA), a sustainable biobased co-monomer, is a suitable choice for fabricating crosslinked bioresin from TO because it offers dual functionality through the furan ring and the acrylate group. Furan ring engages as a diene in the DA reaction with TO, generating an acrylated TO-resin, which can further participate in free radical polymerisation during the curing step to develop the final crosslinked network [13,20]. So far, a number of acrylate-modified TO resins has been reported [10,13,14,21], but most of them are water-resistant TO-alkyd resins, formulated for paint and coating industry [22], and they typically suffer from low tensile strengths (~8–10 MPa) [22], limiting their applications as structural materials. To the best of our knowledge, none of these resins have been explored for their application in shape-memory composites. Often, the major drawback of the shape-memory polymers is low stiffness which results in lower shape recovery rate than metal and ceramics [23]. Incorporation of fillers or reinforcements has been shown to be a viable strategy for improving the mechanical strength of the shape-memory polymers.

Over the past few years, nanocelluloses have extensively used as promising reinforcing agents in composites due to their appealing intrinsic properties, including low density, high surface area, high mechanical strength, renewability and biodegradability [24]. Many reports have demonstrated that incorporation of cellulose nanofiber (CNF) or cellulose nanocrystal (CNC) into shape-memory polymers, such as polyurethane [23,25,26,27] polyvinyl acetate [27] and polyethylene glycol-polycaprolactone [18,27] has led to improved strength and stiffness. However, uniform dispersion of unmodified nanocellulose into hydrophobic matrices is still a challenge due to the hydrophilicity of nanocellulose [28]. Commonly employed physical blending methods often affect the shape-memory performance of the materials [18]. In this context, we hypothesized that direct impregnation of hydrophilic nanocellulose network with the hydrophobic acrylated TO resin would address the compatibility issues [29,30,31,32].

**Scheme 1:**
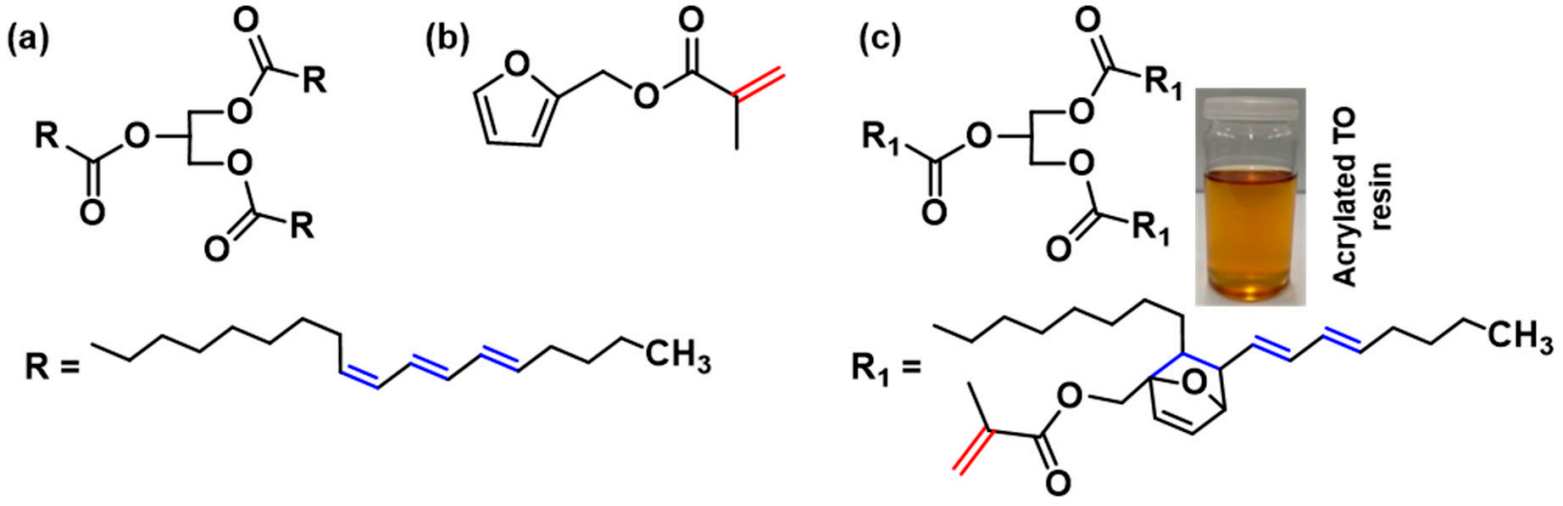
Chemical structure of (**a**) tung oil (TO), (**b**) furfuryl methacrylate (FMA), and (**c**) the probable Diels-Alder (DA) adduct arising from TO and FMA.

In this study, we report fabrication of a new biocomposite with shape-memory behavior from acrylate modified TO bioresin and nanocellulose networks. The structures of TO, FMA and the probable DA adduct are shown in Scheme 1. First, FMA modified TO bioresin was synthesized via the DA reaction. This bioresin was then used to impregnate cellulose nanofiber (CNF) and cellulose nanocrystal (CNC) networks, followed by in-situ curing of the biocomposites. Notably, CNF and CNC were derived from onion skins which has recently been shown to be sustainable source of lignocellulosic fiber with potential application in composite materials [33]. In Europe, every year, >450,000 tonnes of onion skin is generated by food processing industry and common household cooking as a major waste. So far, wood is considered as convenient and main resource for production of high performance nanocellulose, but high demand of wood in many other industries limits its availability with reasonable price in some regions to use it for nanocellulose extraction [34]. This inspired us to explore non-wood resources for producing nanocellulose. Onion skin-waste having high cellulose content (~41%), is a potential cost-effective source of cellulose. In this work, ability of two types of nanocellulose networks (CNF and CNC) to reinforce the synthesized resin was compared. The high aspect ratio (length/diameter) and high crystallinity of CNC networks resulted in a better reinforcing effect than the long flexible CNF networks [35]. We found that nanocellulose reinforcements significantly improved the mechanical strength of the TO-based polymers, and that combinations of nanocellulose networks and TO-based resin are suitable for designing smart hydrophobic and almost transparent biocomposites.

## 2. Experimental Section

### 2.1. Materials

All the reagents used in this work were of reagent grade. Tung oil (light yellow colour, density: 0.937 g/mL at 25 °C, iodine value: >220), furfuryl methacrylate (97%, containing monomethyl ether hydroquinone as inhibitor), hydroquinone (Reagentplus, ≥99%), styrene (stabilized; for synthesis, ≥99%), Luperox^®^P, tert-butyl peroxybenzoate (≤100%), sodium hydroxide (NaOH) pellets, sodium chlorite (NaClO_2_), and sulphuric acid (H_2_SO_4_, 95–97%) were purchased from Sigma-Aldrich Sweden AB (Stockholm, Sweden). For nanocellulose extraction, onion skins were collected from a local restaurant in Borås, Sweden.

### 2.2. Synthesis of Furfuryl Methacrylated TO Resins

Acrylation of TO was performed with FMA via DA reaction [13,14]. Reaction mixtures were prepared using different molar proportions of TO and FMA (1:3, 1:6, and 1:9). Three different acrylated TO resins, T1F3, T1F6, and T1F9 were synthesized, where T and F denote TO and FMA, respectively, and the numbers represent the molar ratios between TO and FMA (1:3, 1:6, and 1:9).

***a) Synthesis of T1F3 resin*:** 10 gm of TO (11.5 mmol) and 5.73 gm of FMA (34.5 mmol) were placed together in a three-necked round-bottom flask and stirred for 5 min with a magnetic stirrer. Before starting the reaction, 0.078 gm of hydroquinone (0.5 wt% of the total weight of reaction mixture) was added to prevent any free radical polymerization between the acrylate groups and TO. The reaction was heated at 150 °C for 5 h under continuous nitrogen flow in the three-necked flask equipped with a reflux condenser. After cooling to room temperature, the product was isolated as a viscous liquid.

***b) Synthesis of T1F6 and T1F9 resins*:** Synthesis of T1F6 and T1F9 resins were carried out following a procedure similar to that of T1F3 resin, using different amount of TO and FMA. For T1F6 resin, 7 gm of TO (8 mmol), 7.98 gm of FMA (48 mmol) and 0.075 gm of hydroquinone were used. For T1F9 resin, 5 gm of TO (5.7 mmol), 8.52 gm of FMA (51.3 mmol) and 0.068 gm of hydroquinone were used.

### 2.3. Extraction of Nanocellulose from Onion Skins and Preparation of Nanocellulose Films

The onion skins were washed with hot water at 80 °C for 2 h to remove dirt and water-soluble polysaccharides. They were then dried in an oven at 100 °C for 24 h. The dried onion skins were milled to a 0.1 mm size powder using a rotor mill (Fritsch Pulverisette14, Fritsch Industries, Idar-Oberstein, Germany). The ground powder was then used for nanocellulose extraction. Two types of nanocellulose, CNF and CNC, were extracted. Alkaline and bleaching treatment was performed as described previously [36]. CNF was obtained by ultrasonicating the bleached cellulose fiber suspension for 1 h with a 130-watt ultrasonic processor (VCX130, Sonics and Materials, Newtown, CT, USA). CNC was obtained by acid hydrolysis of the bleached cellulose fiber using 45% H_2_SO_4_ for 2 h at 45 °C, followed by ultrasonication [33]. The steps used in the preparation of CNF and CNC are shown in Scheme SI1 in the supplementary material.

CNF and CNC films were prepared according to the method described in the literature [36]. Briefly, CNF and CNC suspensions (0.1 wt%) were mixed thoroughly with an IKA-Ultra Turrax digital homogenizer for 10 min at 20,000 rpm. The suspensions were then vacuum filtered using Whatman^®^ polyamide membrane filters (EN 17, pore size 0.45 µm, diameter 47 mm). Residue cakes were then separated from the filter paper and transferred to an acetone bath to remove the residual water and to retain the network structure of nanocellulose. 

Characterizations of CNF and CNC in terms of size, tensile strength, modulus and porosity of films were mentioned in SI document (Table SI1). Porosity of the films were calculated according to Tanpichai et al. [37].

### 2.4. Preparation of Biocomposites

**Scheme 2:**
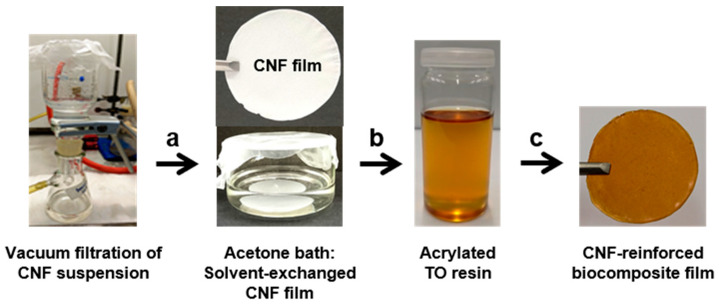
Preparation of the biocomposite films. (**a**) Solvent exchange of the film formed by vacuum filtration, (**b**) impregnation of the solvent-exchanged film with the resin, and (**c**) drying, curing, and post-curing of biocomposite films.

The resin mixtures were prepared by mixing the synthesized acrylated TO (T1F3, T1F6 and T1F9) with 10 wt% styrene and 5 wt% tert butyl peroxybenzoate with respect to total resin weight, for 5 min using a magnetic stirrer. Styrene acts as the crosslinking agent leading to the formation of covalent bonds between the resin molecules [30]. The solvent-exchanged CNF and CNC films were directly transferred to the prepared resin mixture from the acetone bath and the resin mixture was allowed to soak into the CNF and CNC films for 2 h. The biocomposite films were then dried overnight at room temperature. Finally, the dried composite films were cured in a hydraulic hot press (using a 4 × 4 cm mould) at 120 °C for 4 h, and then post-cured at 160 °C for 2 h [38]. The preparation of the biocomposite films is illustrated in Scheme 2. The biocomposites formed are given in Table 1. All the biocomposites contained 10 wt% CNF or CNC content.

### 2.5. Characterization 

The materials used and the final composite films were characterized in several ways. Fourier transform infrared spectroscopy with attenuated total reflectance (ATR-FTIR) was used to obtain spectra from the resins and biocomposites (Nicolet iS10; Thermo Fisher Scientific, MA, USA). The acrylated TO resins and a 1:1 mixture of TO and FMA were analysed with ^1^H and ^13^C NMR using a Bruker DPX 400 spectrometer (Coventry, UK.). CDCl_3_ was used as the solvent for NMR analysis. Scanning electron microscopy (SEM) was performed using a TESCAN MIRA3 FEG-SEM with an SE detector (Everhart–Thornley type, Brno, Czech Republic) at an accelerating voltage of 5 kV to characterize the CNF, CNC suspensions (0.1 wt%) and fracture surfaces of biocomposite films in order to elucidate the interactions between nanocellulose and the resin matrix. Optical microscopy images of the biocomposite films were obtained using a Nikon SMZ800 optical microscope (Amsterdam, the Netherlands) at a magnification of 3.5 ×. Differential scanning calorimetry (DSC) (DSC Q1000; TA Instruments, Newcastle, UK) was used to study the curing behavior of the prepared polymers and biocomposites, and to obtain the glass transition temperature of the biocomposite films. The experiments were carried out in a nitrogen environment at 25–250 °C, and at a heating rate of 10 °C/min. The first heating scans for all the samples were recorded. Dynamic mechanical analysis (DMA) (DMA Q800; TA Instruments, Newcastle, UK) was used to study the thermo-mechanical properties, stress-strain properties of cured resins and biocomposites. Samples of the films were cut into 8 × 5 mm pieces (thickness 0.2 mm). Three samples from each kind of batch were analysed, and the results averaged. Tests were carried out in multi-frequency strain mode to study the thermo-mechanical properties. The frequency was 1 Hz and the temperature range was −20 to 170 °C, at a heating rate of 3 °C/min. Stress-strain measurements were conducted in controlled force mode at 35 °C, using a preload force of 0.01 N and a force ramp of 1 N/min up to 18 N (the maximum force of the machine). To assess the thermal stability of the cured resins and biocomposites thermogravimetric analysis (TGA) (TGA Q1000; TA Instruments, Newcastle, UK) was performed under nitrogen over the temperature range 30‒600 °C at a heating rate of 10 °C/min. Water contact angles of the biocomposite films were measured with an optical tensiometer (Attension Theta, Biolin Scientific, Gothenburg, Sweden) using the sessile drop method. The water droplet volume was 3 µl throughout the measurements. Three random areas were selected on each sample and the average contact angle was determined. Short-term creep tests were conducted on the prepared biocomposites using DMA in tensile mode at different temperatures from 35 °C to 70 °C against a fixed 2 MPa stress. Before each measurement, the samples were equilibrated for 3 min at each temperature to allow the exact temperature to be attained. Creep was allowed to progress for 10 min, and the creep recovery was studied for 20 min. Three rectangular samples (8 × 5 mm) of each batch were analysed, and the average values calculated. UV-Vis spectra were recorded from the CNF and CNC films, and the resin-impregnated composite film samples in transmittance mode using a Cary 60 UV-Vis spectrometer from Agilent (Santa Clara, CA, USA) to characterize the optical transparency of the biocomposites.

## 3. Results and Discussion

**Figure 1:**
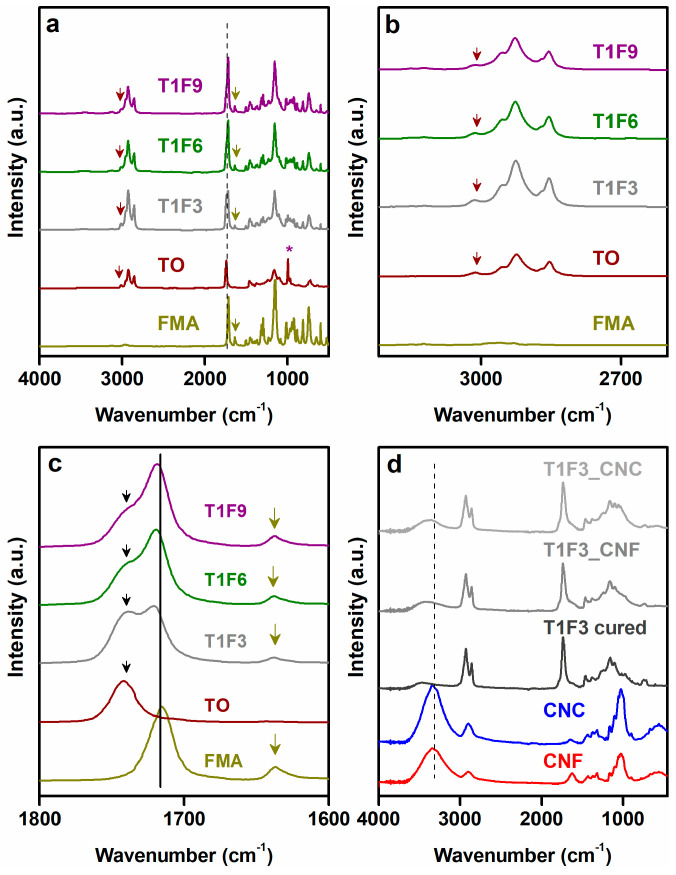
FTIR plots. (**a**) FMA, TO, and synthesized acrylated TO resins. Red and yellow arrows indicate the peaks at 3012 cm^−1^ and 1636 cm^−1^, respectively, the red asterisk (*) indicates the peak at 996 cm^−1^, and the black dotted line indicates the peak at 1720 cm^−1^. (**b**,**c**) show the regions 2600–3250 cm^−1^ and 1600–1800 cm^−1^, respectively (in Figure 1b, the peak at 3020 cm^−1^ was highlighted with red arrow, and, in Figure 1c, the peaks at 1741 cm^−1^, 1720 cm^−1^, and 1635 cm^−1^ are highlighted with black arrow, black solid line, and yellow arrow, respectively). (**d**) cellulose nanofiber (CNF), cellulose nanocrystal (CNC), cured T1F3 resin, T1F3_CNF, and T1F3_CNC biocomposites (black dotted lines indicate the –OH stretching at 3330 cm^−1^). FTIR plots of cured T1F6, T1F9 resins and their composites are shown in Figure SI1.

Biocomposites were fabricated using acrylated TO resins as matrix and onion-skin-derived nanocellulose networks as reinforcements. Resin synthesis was performed by acrylation of TO via Diels-Alder (DA) reaction and the successful reaction was confirmed by ATR-FTIR. In Figure 1a, the FTIR spectrum of TO showed a shoulder peak at 3012 cm^−1^ (Figure 1b) and a strong signal at 996 cm^−1^ corresponding to the *trans-trans* conjugated double bonds, and a weak peak at 964 cm^−1^ arising from the *cis* double bonds (Figure 1a) [39]. The sharp signal at 1742 cm^−1^ was attributed to ester-carbonyl stretching. The other component, FMA, exhibited carbonyl stretching at 1716 cm^−1^, acrylate C=C stretching at 1635 cm^−1^, and furan ring stretching and breathing at 1501 and 1012 cm^−1^. The presence of carbonyl stretching at both 1741 and 1720 cm^−1^ in the synthesized resins (T1F3, T1F6 and T1F9) (Figure 1c) confirmed the incorporation of both raw materials (TO and FMA). Acrylate C=C stretching remained unaltered in the acrylated TO resins, which suggests that it did not take part in the DA reaction. A decrease in the intensities of furan ring stretching and breathing, and the disappearance of TO unsaturation at 996 cm^−1^ suggest that furan ring and TO unsaturation participate in the DA reaction. 

**Figure 2:**
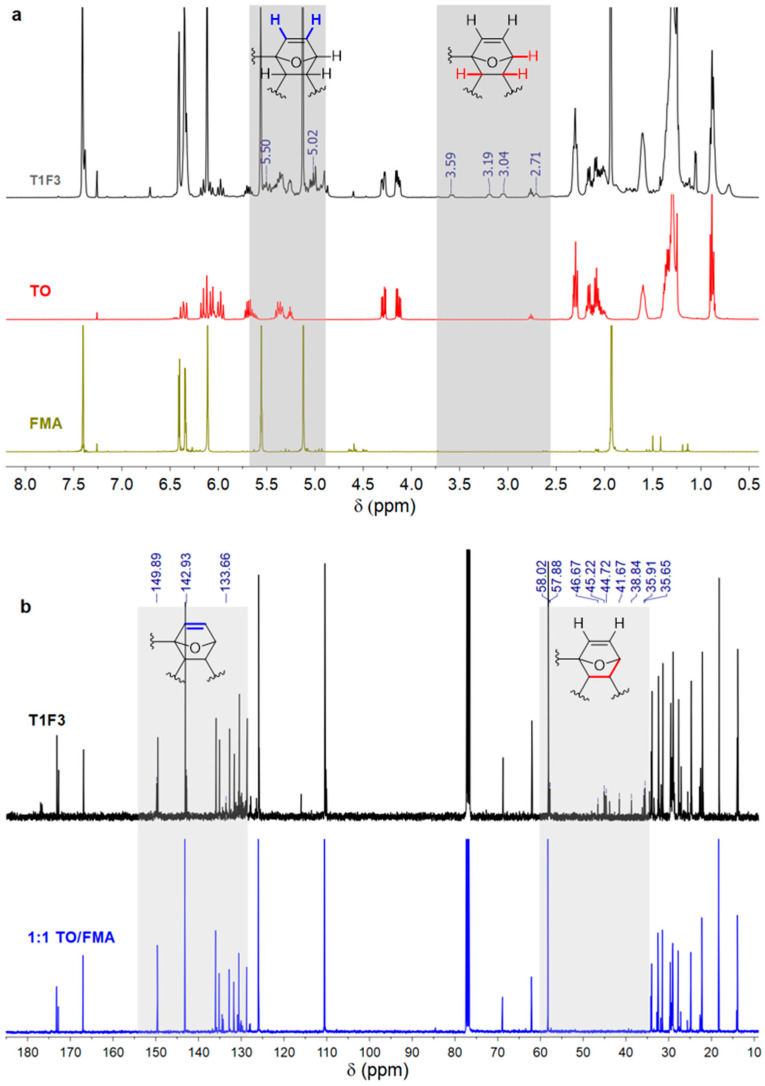
(**a**) ^1^H NMR spectra of FMA, TO, and T1F3 resin. Highlighted grey rectangular shades indicate the regions where new peaks were observed. (**b**) ^13^C NMR spectra of a 1:1 mixture of TO and FMA before reaction, and synthesized T1F3 resin (NMR spectra are only shown for resin T1F3, as the spectra were the same for other two acrylated TO resins, T1F6 and T1F9).

The reaction between the furan ring and TO double bonds was further confirmed by ^1^H and ^13^C NMR analysis of the resins. A tentative structure of the acrylated TO resin is shown in Scheme 1c. ^1^H NMR of the synthesized resin (Figure 2a) revealed new peaks at 3.0–3.6 ppm, which can be attributed to the methine (–CH–) protons in the DA adduct [13,21]. The resonances at ~5.0 and ~5.5 ppm can be assigned to olefinic protons of the newly formed six-membered ring in the resin [40]. The methylene protons adjacent to the triglyceride backbone in TO appear at 4.16–4.40 ppm, and their position remained unchanged in the resin [21]. Comparison of the ^13^C NMR spectrum of the resin to that of a 1:1 mixture of TO and FMA (Figure 2b) revealed new resonances in the aliphatic region, 35–60 ppm, which is consistent with the formation of new saturated carbon centres in the resin via the DA reaction. The NMR spectra also exhibited some extra peaks in the 130–150 ppm range, but it was difficult to distinguish them from the peaks in the starting material, and to conclusively assign them to newly formed C=C groups in the DA adduct. The integration values of methyl protons in T1F3 resin showed (Figure SI2 in the supplementary material) the ratio of TO and FMA was 1:4, which was close to the feed ratio (1:3 for TO:FMA). It is difficult to conclude from the NMR, if all the starting materials have reacted. The product mixture might contain starting materials and the desired product.

The acrylated TO resin was then used to make composites containing CNF and CNC networks as reinforcement. Formation of these CNF and CNC networks were evidenced by SEM analysis. In general, CNCs are rod-like or spherical particles with high aspect ratios, while CNFs are long semi-flexible fibers [41]. In this study, SEM images of onion-skin-extracted CNF and CNC suspensions (0.1 wt%) revealed a porous network of nanosized fibers for both CNF (Scheme SI1a in the supplementary material) and CNC (Scheme SI1b in the supplementary material). Rapid freezing followed by freeze drying of an aqueous CNC suspension during SEM sample preparation promotes the self-assembly of CNCs and the formation of long ultrafine nanofibers via strong hydrogen bonding [42]. On the other hand, the combined effect of acid hydrolysis and ultrasonication helped to break the cells easily in CNC, leading to the formation of much thinner fibers in CNC than in CNF [42,43].

These CNF and CNC networks were then impregnated with the acrylated TO resins to fabricate the biocomposites. Low viscosity of the resins (1.5–2.5 Pa.s at 25 °C) facilitates the diffusion of the resins through the porous network structures of CNF and CNC. It is hypothesized that resin impregnation procedure followed by in-situ curing process of these composites might help to control the moisture absorbance tendency by the nanocellulose networks, which is a major drawback of these kind of natural fiber reinforced biocomposites. In this context, FTIR spectra of the biocomposites were recorded to verify if the surface hydroxyl groups of CNF and CNC networks participate in the chemical reaction during in-situ curing process. The FTIR spectra of CNF and CNC-reinforced biocomposites exhibited peaks similar to those of the cured resins, which indicates weak or no chemical interaction between the nanocellulose and the resin in the biocomposites (Figure 1d and Figure SI1 in the supplementary material). Only the intensity of the surface –OH stretching vibration in the biocomposite films was significantly lower than in the nanocellulose films (CNF and CNC), suggesting that the biocomposites had a reduced tendency to absorb moisture [44,45].

**Figure 3:**
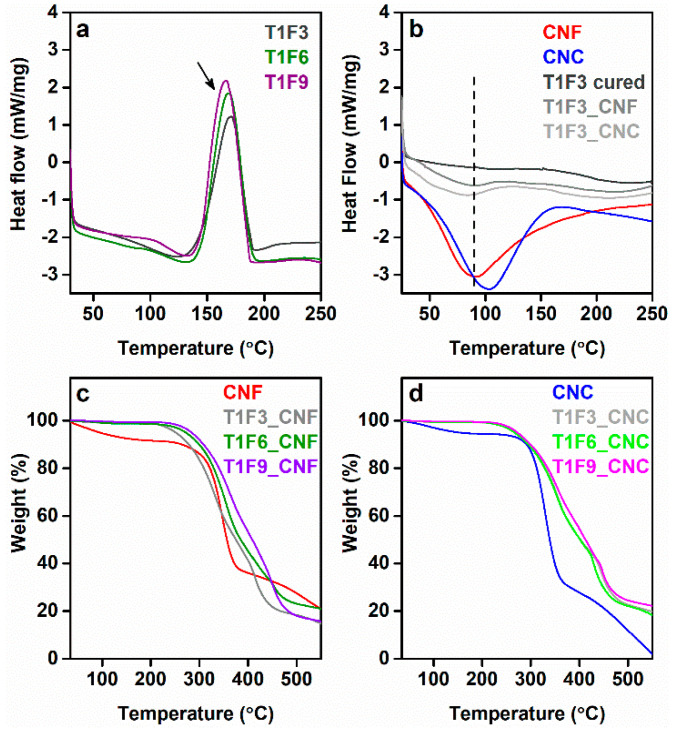
Differential scanning calorimetry (DSC) plots (first heat scans). (**a**) Curing of resins (black arrow indicates the curing reaction temperature at ~165 °C). (**b**) CNF, CNC, cured T1F3 resin, CNF and CNC films impregnated with T1F3 resin (black dashed line indicates the endothermic transitions in CNF and CNC at 30–100 °C). DSC plots of cured T1F6, T1F9 resins, and their composites are shown in Figure SI3 in the supplementary material. Thermogravimetric analysis (TGA) plots of (**c**) CNF and CNF-reinforced biocomposites (T1F3_CNF, T1F6_CNF, and T1F9_CNF), (**d**) CNC, and CNC-reinforced biocomposites (T1F3_CNC, T1F6_CNC, and T1F9_CNC). TGA plots of cured resins are shown in Figure SI4 in the supplementary material.

DSC was performed to study the effects of curing the biocomposites and resins. Curing study of the resins showed sharp exothermic transitions at ~165 °C (Figure 3a), which is attributed to free radical curing. The cured resins (T1F3, T1F6, and T1F9) showed two broad endothermic transitions in the ranges 40–100 °C and 160–220 °C (Figure SI3a in the supplementary material). This indicates the possibility of two glass transition temperatures (*T*_g_) in the materials: one at a lower temperature (~50 °C) due to the long hydrocarbon chain (a soft segment), and the other at a higher temperature (~175 °C) originating from the crosslinked network in the resins (a rigid segment) [10,21]. The soft segment can move easily with increasing temperature, while the hard segment is more rigid. The presence of two glass transition regions suggests a phase separation in the structure of the cured resins [21]. The broad endotherms could be attributed to the enthalpy relaxation process near *T*_g_s. The absence of any exothermic transition in the cured resins and biocomposites indicates complete curing of the samples. The presence of sharp endothermic transition between 90–100 °C in CNF and CNC (Figure 3b) indicates the evaporation of moisture absorbed by the surface hydroxyl groups of CNF and CNC. After impregnation with the resin, small endothermic humps were observed in the same region, consistent with a reduced tendency of the biocomposite films to absorb moisture (Figure 3b and Figure SI3b in the supplementary material). The presence of two glass transition regions in the biocomposites, similar to the cured resins, indicates that the incorporation of nanocellulose networks into the resin did not affect the thermal properties of the resins.

The thermal stability of the resins and composites was assessed by TGA, and the results are given in Figure 3c,d and Figure SI4 in the supplementary material. The cured acrylated TO resins were thermally stable up to 210 °C (Figure SI4 in the supplementary material). Further heating led to degradation of the resins in three steps: (a) decomposition of unreacted components at 250–340 °C, (b) degradation of the crosslinked polymer structure at 340–490 °C, and (c) slow degradation of the resin residue at >490 °C [13]. Incorporation of the CNF or CNC network into the resin thus had no significant influence on the thermal decomposition of resins, in agreement with previous findings by Azizi Samir et al. [46]. CNF and CNC films showed a small weight loss (~8% for CNF and ~5% for CNC) up to a temperature of 150 °C, due to moisture loss, whereas the weight of the biocomposite films was stable up to 210 °C (Figure 3c,d), due to lower moisture absorption by the biocomposites [47]. To support the hydrophobicity of the biocomposite films, relative water absorption capacity (RWAC) of the samples were recorded [48,49]. While CNF or CNC films showed RWAC 240% and 170%, respectively, after 24 h, the RWAC values for CNF and CNC biocomposites were only 3% and did not change even after 5 days.

**Figure 4:**
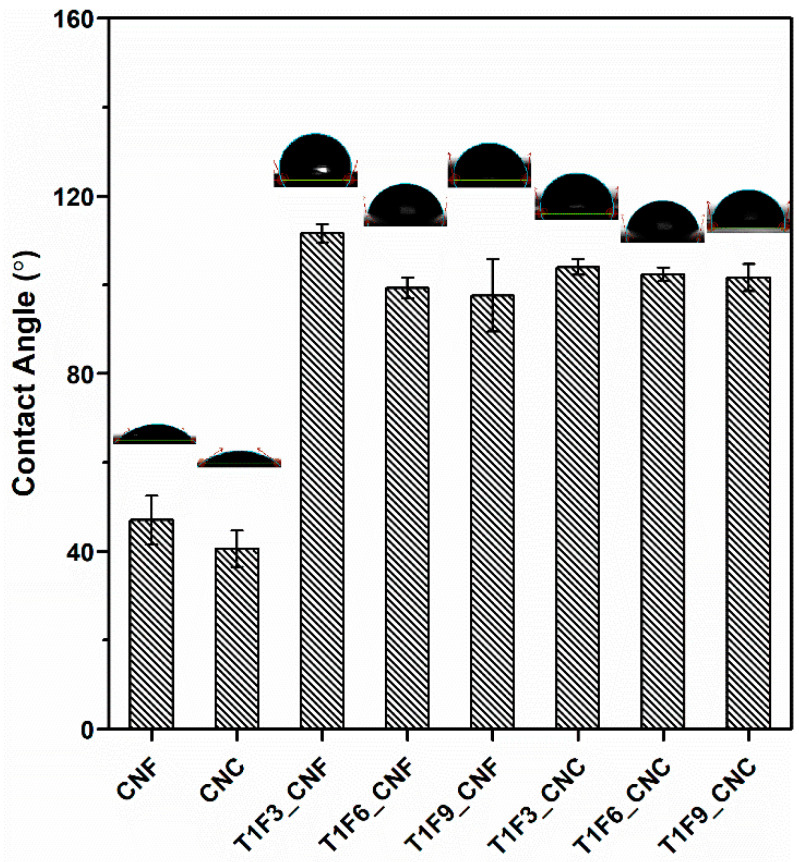
Contact angle plots and water droplet images for CNF, CNC, CNF-reinforced (T1F3_CNF, T1F6_CNF, and T1F9_CNF), and CNC-reinforced biocomposite films (T1F3_CNC, T1F6_CNC, and T1F9_CNC).

Contact angle measurements of the materials showed a significant increase in the degree of hydrophobicity of the biocomposite films compared with CNF and CNC, as can be seen in Figure 4. The relatively low contact angles of CNF and CNC are the result of the hydrophilic nature of the surface of cellulose. Thus, impregnation of the CNF and CNC films by the resin followed by in-situ curing limits the number of hydroxyl groups accessible on the surface rendering the surface of the biocomposites hydrophobic [50,51]. This is consistent with the hydrophobic nature of TO-based resins (contact angle ~100°) [52]. The contact angles were slightly smaller in T1F6_CNF/CNC and T1F9_CNF/CNC than in T1F3_CNF/CNC due to the rough surfaces and inhomogeneity of the samples (T1F6_CNF/CNC and T1F9_CNF/CNC).

**Figure 5:**
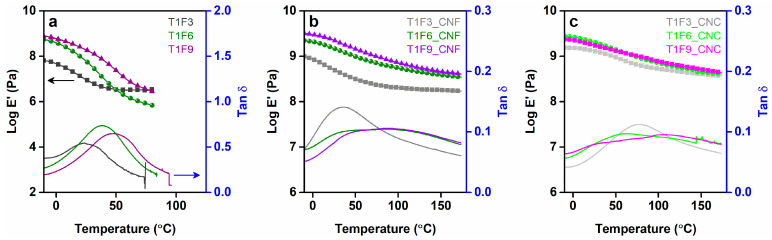
Storage modulus and tan δ plots from dynamic mechanical analysis. (**a**) Cured resins (T1F3, T1F6, and T1F9), (**b**) CNF-reinforced biocomposites (T1F3_CNF, T1F6_CNF, and T1F9_CNF), and (**c**) CNC-reinforced biocomposites (T1F3_CNC, T1F6_CNC, and T1F9_CNC).

DMA was performed to gain insight into the thermo-mechanical behavior of the cured resins and the composite materials. It can be seen from Figure 5a that in the glassy state, the resins with the higher FMA contents (T1F6 and T1F9) have a higher storage modulus than that with the lower FMA content (T1F3). Higher FMA contents lead to increased steric bulk due to the DA adducts, yielding stiffer resins [13,53]. A sharp decrease in the storage modulus was observed in the range −10 °C to 20 °C for T1F3 and −10 °C to 40 °C for T1F6 and T1F9, due to the increase in segmental mobility of the long flexible hydrocarbon chains from the acrylated TO moiety. The rubbery plateau observed at >20 °C for T1F3, and at >40 °C for T1F6 and T1F9 indicates the presence of a stable crosslinked network in all the cured resins [11,12,54]. During curing of the resins, styrene molecules react with the acrylated TO molecule via free radical polymerization, leading to a crosslinked polymer network. The crosslinking densities of the cured resins and composites can be calculated (Table 2) by rubber elasticity theory using the expression: $E^{\prime }=3nRT$, where E′ is the storage modulus in the rubbery region, n is the crosslinking density, R is the universal gas constant and T is the absolute temperature in K (*T* = *T*_g_ + 398 K) [12,54]. Low crosslinking densities in the cured resins caused the films to tear at temperatures above 80 °C due to the low strength of the materials [55]. Incorporation of the CNF and CNC networks into the resins as reinforcement led to marked improvements in the storage modulus (Figure 5b,c) in both the glassy and rubbery regions. The mechanical integrity of the nanocellulose networks is preserved throughout the glassy and rubbery regions, indicating strong reinforcement of the resin matrices [30]. Resin composition also influenced the thermo-mechanical properties of the biocomposites, as demonstrated by the increase in storage modulus with increasing FMA content (Figure 5b,c).

**Figure 6:**
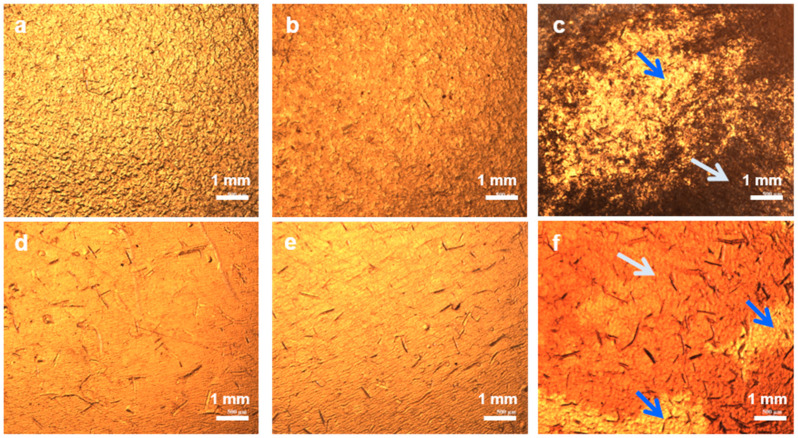
Optical microscopy images of CNF- and CNC-reinforced biocomposites. (**a**) T1F3_CNF, (**b**) T1F6_CNF, (**c**) T1F9_CNF, (**d**) T1F3_CNC, (**e**) T1F6_CNC, and (**f**) T1F9_CNC. (The two different phases: the lighter (soft) phase and the darker phase (rigid) are indicated by the blue and white arrows, respectively.)

Tan δ signals provided the information regarding viscoelastic damping of the materials (Figure 5a–c). Significant decreases in tan δ values (~75–87%) were observed for both the CNF- and CNC-reinforced biocomposites. Interfacial interactions between the polymer matrix and the CNF and CNC networks restrict the movement of polymer chains, causing increased elasticity of the composites. Consequently, less energy is dissipated during mechanical vibration of these materials [35]. As can be seen from Figure 5, all the resins, and the T1F3 biocomposites (both CNF and CNC) display a single broad tan δ transition, whereas the T1F6 and T1F9 biocomposites show broad tan δ transitions with two overlapping peaks (*T*_g_ ~40–60 °C and ~90–100 °C). This implies the presence of two different polymer phases in the resins and biocomposites consisting of soft and rigid segments [30,52,56], which is in agreement with the results obtained with DSC. The phase separation is more prominent in T1F9_CNF/CNC biocomposites, as demonstrated by optical microscopy, where dark and light regions are clearly visible in T1F9 biocomposites (Figure 6).

**Figure 7:**
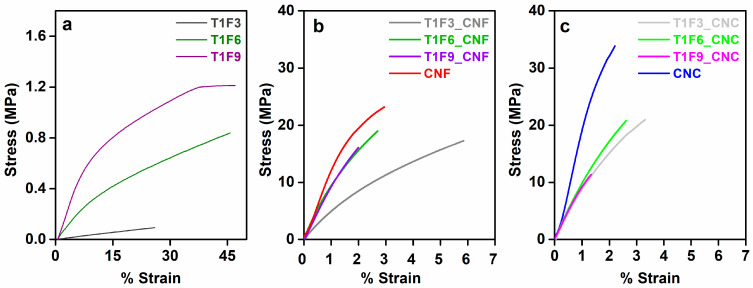
Stress-strain plots obtained from dynamic mechanical analysis. (**a**) Cured resins (T1F3, T1F6, and T1F9), (**b**) CNF-reinforced biocomposites (T1F3_CNF, T1F6_CNF, and T1F9_CNF), and (**c**) CNC-reinforced biocomposites (T1F3_CNC, T1F6_CNC, and T1F9_CNC).

DMA experiments were performed in controlled force mode to investigate the flexibility of cured resins and biocomposite films. As can be seen from Figure 7a, acrylated TO resins are extremely soft and stretchable elastomeric materials with very low strength and high yield strain. Weak intermolecular forces and the less crosslinked structure are mainly responsible for the elastomeric nature of these resins [57,58,59]. Increasing the rigid moieties (FMA content) in the resins restricts the movement of the flexible hydrocarbon chain of TO, leading to increased strength of the resin (T1F9 > T1F6 > T1F3) [22]. Incorporation of the nanocellulose networks into the resins improved the strength of the resins significantly. The higher crystallinity of CNC, together with its extensive intermolecular interactions, result in higher mechanical strength than CNF, i.e., CNC has a better reinforcing effect in the biocomposites. Flexibility of the biocomposite films is affected by two factors: (i) the ordered structure of CNC (Figure SI5 in the supplementary material) and (ii) the resin composition. The first factor dominates in T1F3_CNC/CNF biocomposites, while the second factor dominates in the other biocomposites with higher FMA contents (T1F6_CNF/CNC and T1F9_CNF/CNC) [51]. CNF and CNC-reinforced T1F6 and T1F9 biocomposites exhibit low strength due to the non-homogeneity of the samples caused by phase separation during the in-situ curing process.

**Figure 8:**
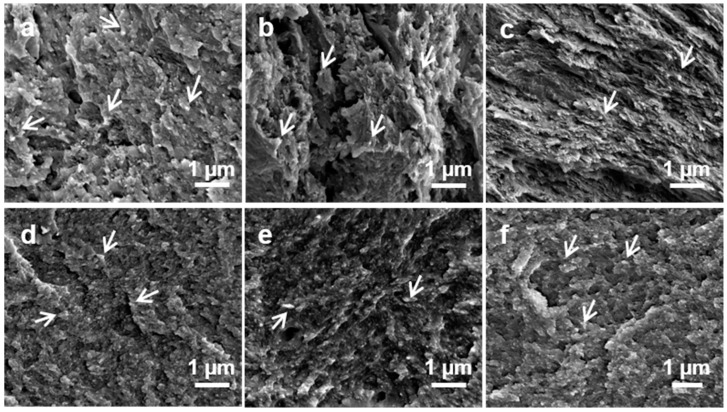
SEM images of fracture surfaces of (**a**) T1F3_CNF, (**b**) T1F6_CNF, (**c**) T1F9_CNF, (**d**) T1F3_CNC, (**e**) T1F6_CNC, and (**f**) T1F9_CNC. (broken and short individual fibre ends are shown with white arrows. All the images were obtained using 20 kx magnification and a 5 kV accelerating voltage.)

Figure 8 shows fracture surface images of biocomposite films. Both CNF and CNC films showed compact, dense network structures (Figure SI6 in the supplementary material) containing mixed-sized fibers, which could be the result of vacuum filtration during film preparation [60]. The high degree of dispersion of the nanofibers reduced the filtration rate and allowed the nanofibers to self-assemble and form a compact network [61]. In the CNF and CNC-reinforced biocomposite films, broken, extremely short individual nanofibril ends were seen as white dots in the SEM images (indicated by white arrows in Figure 8a–c,d–f). This indicates homogeneous distribution of the CNF and CNC fibers throughout the matrix and good adhesion between the resins and fiber network [30]. T1F6_CNF/CNC and T1F9_CNF/CNC showed layered structures corresponding to aggregated CNF or CNC films [56]. This illustrates the non-uniformity of the samples, consisting of a nanocellulose-rich part and a resin-rich part. Furthermore, T1F6_CNF/CNC and T1F9_CNF/CNC had much rougher surfaces than T1F3_CNF/CNC, indicating brittle fracture failure. The surface roughness increased with increasing FMA content in the resin, which might be due to inhomogeneity in the samples.

**Figure 9:**
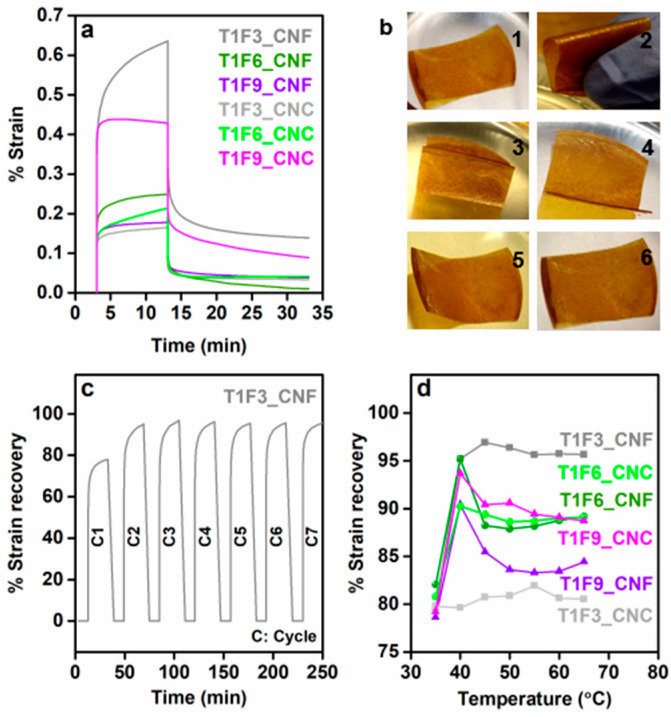
(**a**) % creep strain vs. time plots for CNF- and CNC-reinforced biocomposites at room temperature, (**b**) different stages of shape recovery of a T1F3_CNF biocomposite film (**1:** normal shape of biocomposite film at 30 °C, **2:** hand-folded film at 30 °C, **3** and **4:** slow shape recovery at 30 °C, **5** and **6:** fast shape recovery to original shape upon heating to 50 °C), (**c**) % strain recovery vs. time plot for a T1F3_CNF biocomposite film obtained with a cyclic creep test and (**d**) % strain recovery at different temperatures for CNF- and CNC-reinforced biocomposites.

The prepared biocomposites exhibited interesting shape recovery properties. The shape-memory behavior of a CNF-reinforced biocomposite film is shown in a video clip attached as Video SI. At room temperature, a hand-folded biocomposite film exhibited very slow recovery, whereas the deformed film regained its original shape much more quickly upon heating. Short-term creep measurements were performed to characterize this shape recovery behavior at different temperatures. Figure 9a shows the strain vs. time plots for different CNF and CNC biocomposites at room temperature. Resin composition had a strong influence on the creep properties, as well as on the shape recovery of the materials (shape recovery of neat T1F3 resin was shown in Figure SI7 in the supplementary material), and, as shown in Figure 9a, the % creep strain decreased with increasing FMA content in the CNF composites. However, the opposite effect was seen in the CNC composites, i.e., the % creep strain increased with increasing FMA content at room temperature. The increased steric bulk in the resin structure and the stiffness imparted by the dense network of the CNF likely restrict the reorientation and mobility of the polymer chains, thus reducing the % creep strain in the CNF-reinforced biocomposites in the order: T1F3_CNF > T1F6_CNF > T1F9_CNF [62]. In contrast, the less dense structure and high crystallinity of the CNC network are less effective in restricting the slippage of polymer chains [62,63]. This allows more dislocations in the samples, resulting in enhanced creep deformations of the CNC biocompositesin the order T1F3_CNC < T1F6_CNC < T1F9_CNC. The % creep strain increased with temperature in all the samples (Figure SI8 in the supplementary material). Figure 9b shows photographs of the different stages of shape recovery of a T1F3_CNF biocomposite film. The stress-strain cyclic result derived from the creep measurement was used to qualitatively explain the shape recovery behavior. A representative time-dependent % strain recovery of T1F3_CNF is shown in Figure 9c, showing 96% strain recovery after the 7th cycle. Between 80 and 96% strain was recovered after the 7th test cycle in all the CNF and CNC composites in the temperature range 35–70 °C (Figure 9d). Crosslinking has the strongest influence on the final creep and shape recovery properties of the materials. Higher crosslinking points help to store more elastic deformation energy in the material, which is released upon heating, allowing the material to regain its original shape [64,65]. The incorporation of CNC and CNF networks increases the crosslinking, leading to an increase in the rigid segments in the biocomposites, which are primarily responsible for permanent shape and shape recovery in the materials [66,67]. Most samples exhibited the best shape recovery close to the glass transition temperatures (Figure 9d), due to activation of frozen micro-Brownian motion of the molecular chains [67,68].

**Figure 10:**
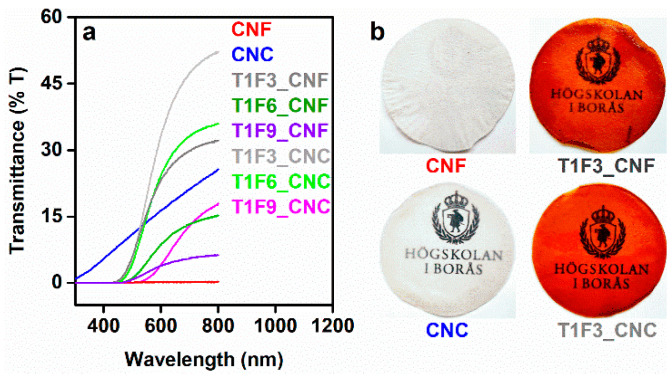
(**a**) Light transmittance spectra obtained from CNF, CNC, and their biocomposites. (**b**) Transparency images of CNF, CNC, T1F3_CNF, and T1F3_CNC films.

UV-Vis transmittance spectra obtained from the samples revealed that T1F3 biocomposites had higher transparency (32% and 52% transmittance at 800 nm for T1F3_CNF and T1F3_CNC, respectively) than other composites with higher FMA contents (Figure 10a). Overall, the CNC composites showed higher transmittance than the CNF composites. Smaller nanoparticle sizes, lack of agglomeration and lack of entanglement between fibers in CNC facilitate its homogeneous impregnation by the resin, which led to the formation of almost optically transparent CNC composite films (Figure 10b) [35,69].

Increasing the FMA content in the biocomposites increased the concentration of chromophores (furans) in the material, leading to more coloured and opaque films (Figure SI9a,b in the supplementary material). Biocomposite films showed strong absorptions over a broad range covering UV (300–400 nm) and visible regions (400–700 nm) (Figure SI9c in the supplementary material), explaining the intense yellow colour of the materials. These absorptions could be assigned to the unsaturations present in acrylated TO resins and styrene. The zero transmittance of UV light up to 400 nm for all the biocomposite films make them suitable as good UV barrier materials [70]. 

In comparison to metals and ceramics, low strength and stiffness of the shape-memory polymers often affect their shape-recovery property. Thus, addition of fillers or reinforcements to these polymers is required to increase the stiffness, as well as recovery force to meet certain application needs [23]. Earlier reports have shown that incorporation of cellulose reinforcements (CNC, CNF, and wood flour) into polyurethane-based shape-memory composites led to a modest increase in strength (<2-fold) and storage modulus (<5-fold) over neat polymers [25,26,71,72,73]. In contrast, we demonstrate that in our case, the onion-skin-derived nanocellulose networks (CNF and CNC) improved the strength and storage modulus of the neat polymers by >192-fold and >30-fold, respectively, without affecting the shape-recovery property and the ductility of the composites. This indicates a significant upgrade to previously reported materials.

## 4. Conclusions

Furan derivative- (FMA) and vegetable oil- (TO)-based bioresins were synthesized via the Diels-Alder reaction with various TO:FMA ratios. Food-waste (onion skin)-derived nanocellulose networks (CNF and CNC) were incorporated into the bioresins to fabricate biocomposites, which exhibited significantly improved storage modulus, strength (190–240-fold increase in mechanical strength) and increased hydrophobicity. The incorporation of CNC networks led to greater improvements in the properties of the biocomposites due to its high degree of crystallinity and less dense structure in comparison to CNF. Increasing the FMA content in the resins led to phase-separated, brittle biocomposite films with poor transparency, suggesting that the properties of these composite materials could be tuned by altering the ratios of the raw materials. Notably, the biocomposite films exhibited interesting shape-memory behavior resulting from the presence of soft and rigid segments due to the acrylated TO resin. A significant degree of shape recovery (~80–96%) was observed in the biocomposites, which is comparable to shape recovery properties of polyurethane. This study demonstrates a convenient method for fabricating lightweight, flexible, and hydrophobic shape-memory composite materials with tailored properties in a sustainable manner using renewable components, household food wastes, and a clean synthesis protocol. These types of materials are in high demand in packaging, smart textile, biomedical, and electronic industries, where thermally activated shape-memory properties are required.

## Figures and Tables

**Table 1:** Compositions and nomenclature of the resins and biocomposites used.

Resins	TO:FMA Mole Ratio	10% CNF-Reinforced Biocomposites	10% CNC-Reinforced Biocomposites
T1F3	1:3	T1F3_CNF	T1F3_CNC
T1F6	1:6	T1F6_CNF	T1F6_CNC
T1F9	1:9	T1F9_CNF	T1F9_CNC

**Table 2:** Summary of mechanical and thermal properties of cured resins, as well as their CNF and CNC-reinforced biocomposites.

Sample	E′ at 25 °C (MPa) ^a^	*T*_g_ (°C) ^b^	Loss Factor (Tan δ)	n (10^3^ mol m^−3^) ^c^	Strength (MPa)	Tensile Modulus (MPa) ^d^	*T*_i_ (°C) ^e^	*T*_50_ (°C) ^e^
**T1F3**	15	25	0.54	1.49	0.09	0.34	262	410
**T1F6**	63	39	0.73	2.98	0.84	1.83	259	399
**T1F9**	199	49	0.64	5.59	1.2	3.08	259	402
**T1F3_CNF**	490	36	0.14	100	17.28	288	217	375
**T1F6_CNF**	1492	41; 94	0.1	305	19.01	637	230	385
**T1F9_CNF**	2250	52; 95	0.1	442	16.09	805	255	408
**T1F3_CNC**	1317	77; 105	0.11	206	20.85	630	242	402
**T1F6_CNC**	1723	58; 110	0.09	300	21	780	239	402
**T1F9_CNC**	2047	32; 107	0.09	322	11.42	814	248	408

^a^ E′: storage modulus, ^b^ *T*_g_: glass transition temperature from dynamic mechanical analysis (DMA), ^c^ n: crosslinking density, ^d^ Tensile modulus were calculated from stress-strain plot. ^e^ *T*_i_: initial degradation temperature from TGA, ^e^ *T*_50_: 50% weight loss temperature from TGA.

## Supplementary Materials

The following are available online at https://www.mdpi.com/2073-4360/12/11/2470/s1, Scheme SI1. Extraction of CNF and CNC from onion skins and SEM images of aqueous CNF and CNC suspensions (0.1 wt%). Table SI1. Characterization and properties of CNF and CNC. Figure SI1. FTIR plots of cured T1F6, T1F9 resins and their CNF/CNC composites. Figure SI2. NMR spectrum of T1F3 resin with integration values of peaks Figure SI3. DSC plots of cured resins and biocomposite films (T1F6_CNF/CNC and T1F9_CNF/CNC). Figure SI4. TGA plots of cured resins. Figure SI5. XRD plots of CNC, CNF and T1F6_CNF/CNC biocomposite films and optical microscopy images of T1F6_CNF/CNC biocomposite films at high magnification. Figure SI6. SEM images of fracture surfaces a) CNF and b) CNC film. Figure SI7. Different stages of shape recovery of neat resin T1F3 film. Figure SI8. Creep test results for CNF- and CNC-reinforced biocomposites at different temperatures. Figure SI9. Photographs of opaque T1F9_CNF/CNC biocomposite films and UV-vis absorption spectra of biocomposites. Video SI. Shape recovery of a CNF-reinforced biocomposite film (T1F3_CNF).
