# Supplementary material for: Hydrophobic Shape-Memory Biocomposites from Tung-Oil-Based Bioresin and Onion-Skin-Derived Nanocellulose Networks
Source: Polymers (Basel). 2020 Oct 25;12(11):2470. doi: 10.3390/polym12112470 (PMC7716223; doi:10.3390/polym12112470)
Supplement: Supplementary file 1 [file polymers-12-02470-s001.pdf]

Supplementary Information (SI)

Hydrophobic shape-memory biocomposites from tung-oil-based bioresin and onion-skin-derived nanocellulose networks

Sunanda Sain[1], Dan Åkesson[1*], Mikael Skrifvars[1], Souvik Roy[2]*

[1]Swedish Centre for Resource Recovery, University of Borås, SE-501 90 Borås, Sweden

[2]School of Chemistry, University of Lincoln, Joseph Bank Laboratories, Lincoln, LN6 7DL, UK

*Correspondence: sunanda.sain@hb.se (S.S.); dan.akesson@hb.se (D.Å.)

Nanocellulose extraction from onion skin and characterizations

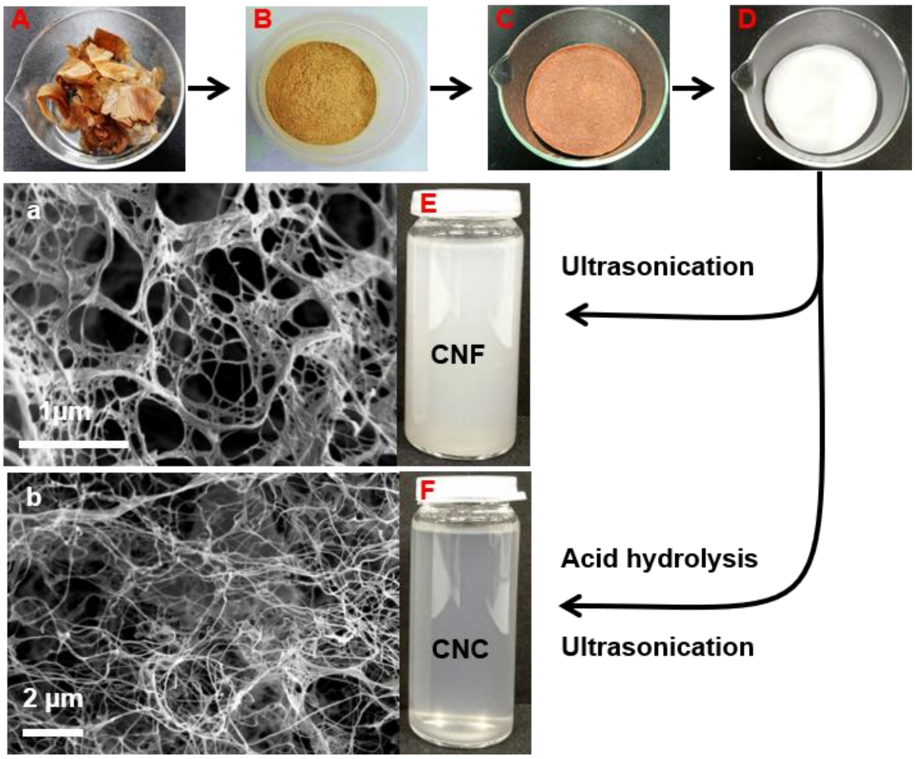

Scheme SI1. Extraction of CNF and CNC from onion skin. (A) Washed onion skin, (B) ground onion skin powder, (C) 2% NaOH treated onion skin powder, (D) bleached onion skin powder, (E) 0.1 wt% CNF suspension, (F) 0.1 wt% CNC suspension. SEM images of a) CNF (75kx) and b) CNC (20kx) suspensions (0.1 wt%).

Formation of CNF and CNC network structures from onion skin was verified through SEM analysis of the 0.1 wt% CNF and CNC suspensions. SEM samples were prepared by freezing followed by freeze drying of a small drop of sample placed on a carbon tape.

Extracted CNF and CNC were characterized using SEM, XRD and porosity of the CNF and CNC films were calculated using the following equation:

$$\varepsilon\% = \left[1 - \left({}^{\rho_f}/_{\rho_{cellulose}}\right)\right] \times 100$$

ε is the porosity of the film, ρ_f is the density of the film, which is calculated from weight of the film and volume of the film. $\rho_{cellulose}$ is the density of cellulose (1.5 g cm^{-3}). [Tanpichai et al.,2019- ref 37 from main manuscript] The average porosity value of five films from each set are reported.

The approximate average size of CNF and CNC were measured from SEM through image processing software (different regions were selected for length and diameter measurement and the average values were reported). Tensile strength and modulus were calculated from stress-strain plots.

Table SI1: Characterization and properties of CNF and CNC

Sample	Length (L) (μm)	Diameter (D) (nm)	Aspect ratio (L/D)	Porosity of films (%)	Tensile strength (MPa)	Tensile modulus (MPa)
CNF	1.93	69	28	82.7	23.4	793
CNC	4.09	58	70	76.2	34.1	1550

FTIR

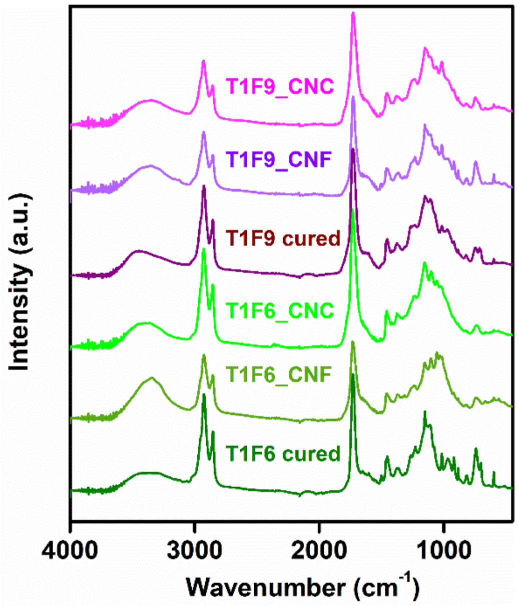

Figure SI1. FTIR plots of cured T1F6, T1F9 resins and their CNF and CNC-reinforced biocomposites.

NMR

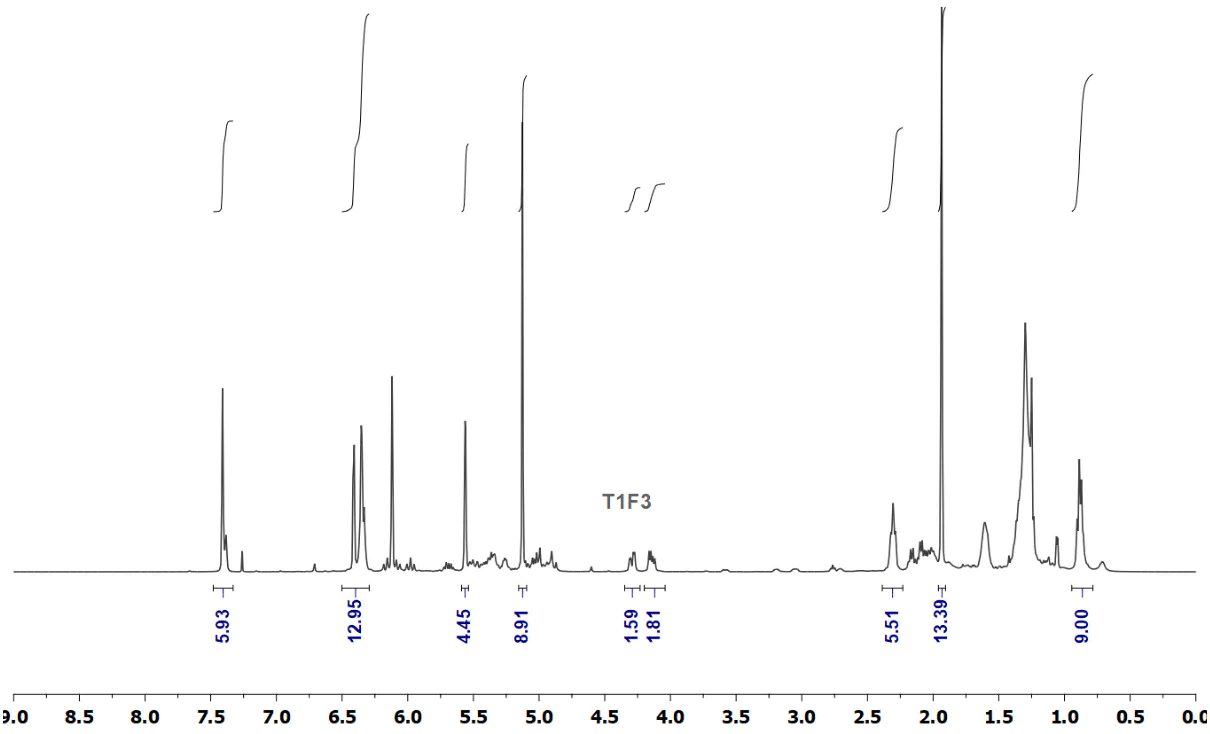

Figure SI2. NMR spectrum of T1F3 resin with integration values of peaks (the terminal methyl groups from TO at 0.89 ppm, and the methyl group from FMA at 1.98 ppm were integrated for determining the ratio).

DSC

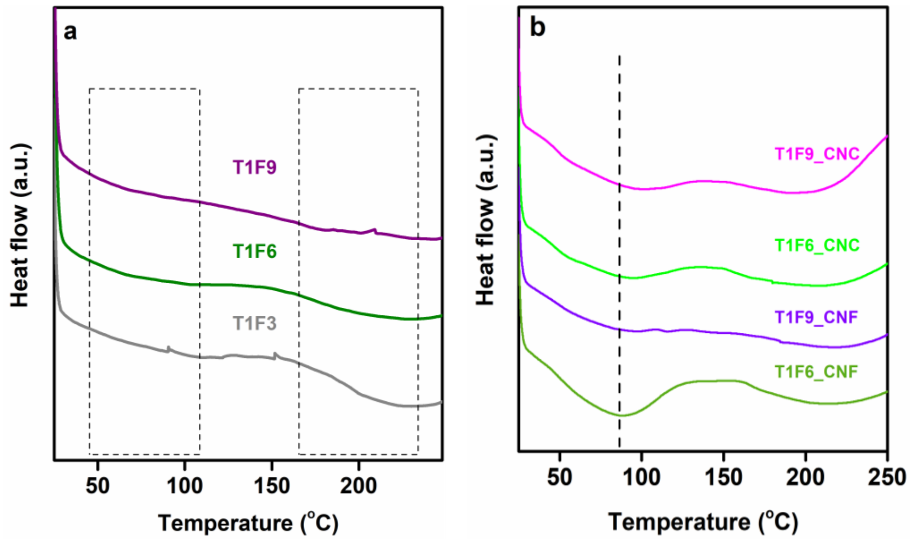

Figure SI3. DSC plots of a) cured resins (T1F3, T1F6 and T1F9) (black dashed rectangles indicate the endothermic transition regions at 40–100 °C and 160–220 °C), b) T1F6_CNF/CNC and T1F9_CNF/CNC biocomposites.

TGA

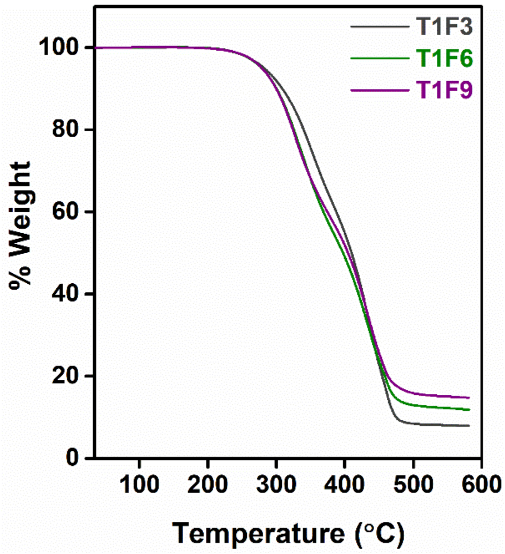

Figure SI4. TGA plots of cured resins (T1F3, T1F6 and T1F9)

XRD

X-ray diffractometry (XRD) pattern of CNF, CNC and biocomposite films were collected on a Panalytical Empyrean diffractometer (Cu radiation) [Malvern Panalytical Ltd., Malvern, UK.] to determine the crystallization property of the materials. The samples were scanned from 2θ = 5° to 2θ = 60°.

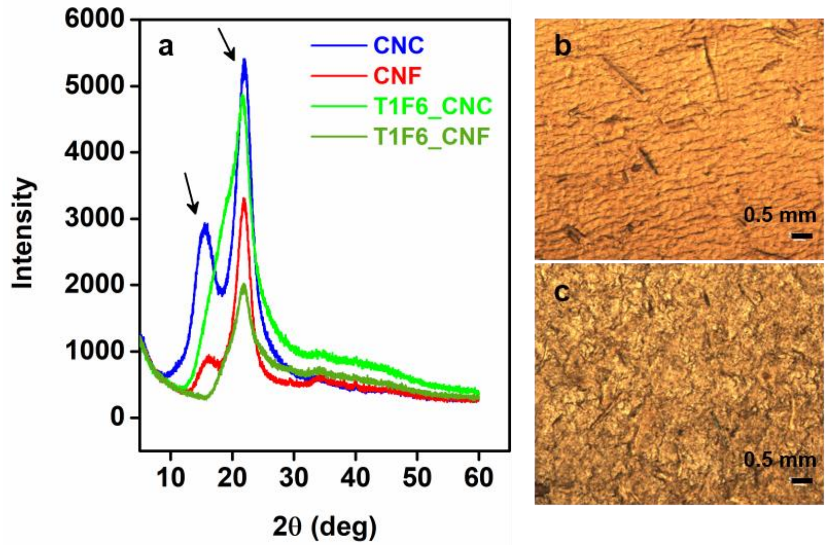

Figure SI5. a) XRD plots of CNC, CNF, T1F6_CNF and T1F6_CNC biocomposite films. The black arrows indicate the peaks at 2θ =16.2° and 22°) and Optical microscopy images of b) T1F6_CNC and c) T1F6_CNF biocomposite films (magnification 5.0×)

From XRD plots higher crystallinity was observed for CNC compared to CNF. Similarly, CNC reinforced biocomposite showed higher crystalline character (T1F6_CNC and T1F6_CNF were shown as examples) compared to CNF reinforced biocomposite. Ordered structure of T1F6_CNC biocomposite film was also observed in optical microscopy image in Figure SI5b.

SEM: Fracture surface images of CNF and CNC film

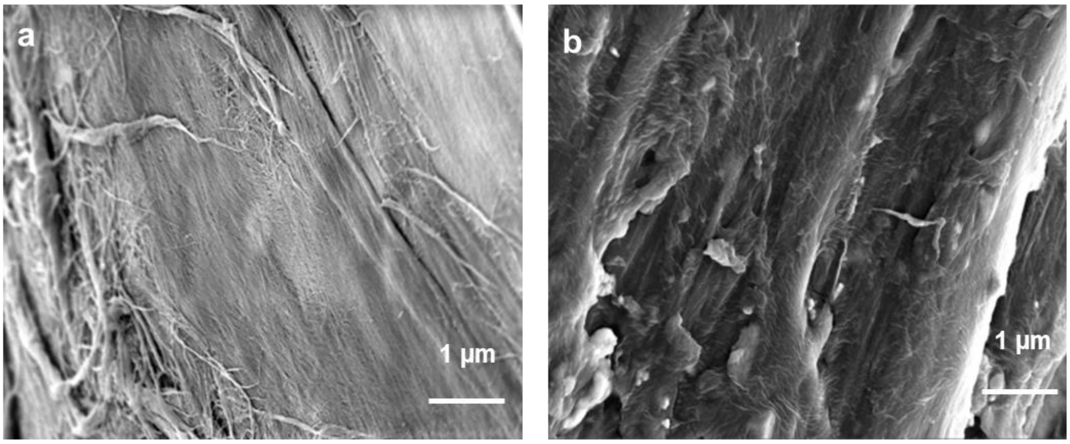

Figure SI6. SEM images of fracture surfaces a) CNF and b) CNC film (images were obtained using 20 kx magnification and a 5 kV accelerating voltage)

Shape recovery images of neat resin (T1F3):

Neat resin film (T1F3) showed shape recovery behaviour at room temperature. Resin structure with crosslinked part and long hydrophobic chain might be responsible for exhibiting this shape memory behaviour.

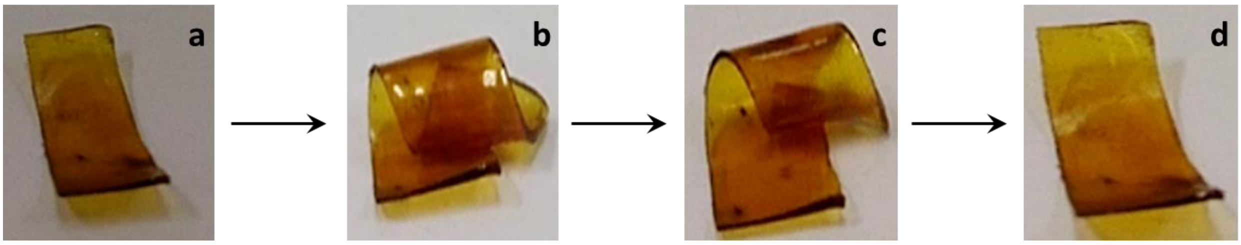

Figure SI7. Different stages of shape recovery of neat resin T1F3 film at room temperature 30 °C (a: normal shape of resin film, b: hand-folded film, c: shape recovery, d: shape recovery to original shape)

Creep test

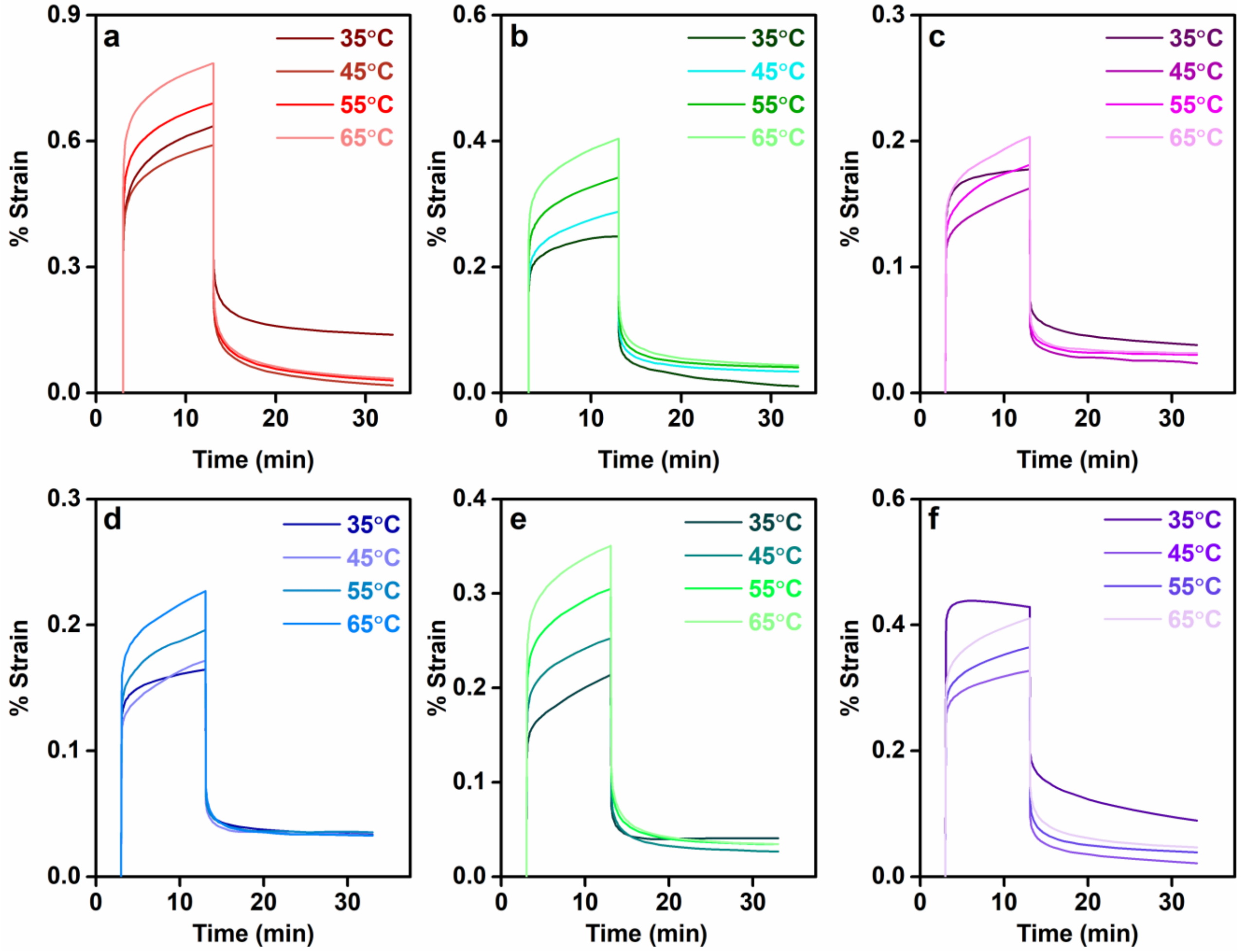

Figure SI8. %Creep strain and recovery against time at different temperatures (35, 45, 55 and 65 °C)) for CNF and CNC-reinforced biocomposites. a) T1F3_CNF, b) T1F6_CNF, c) T1F9_CNF, d) T1F3_CNC, e) T1F6_CNC and f) T1F9_CNC.

Transparency test

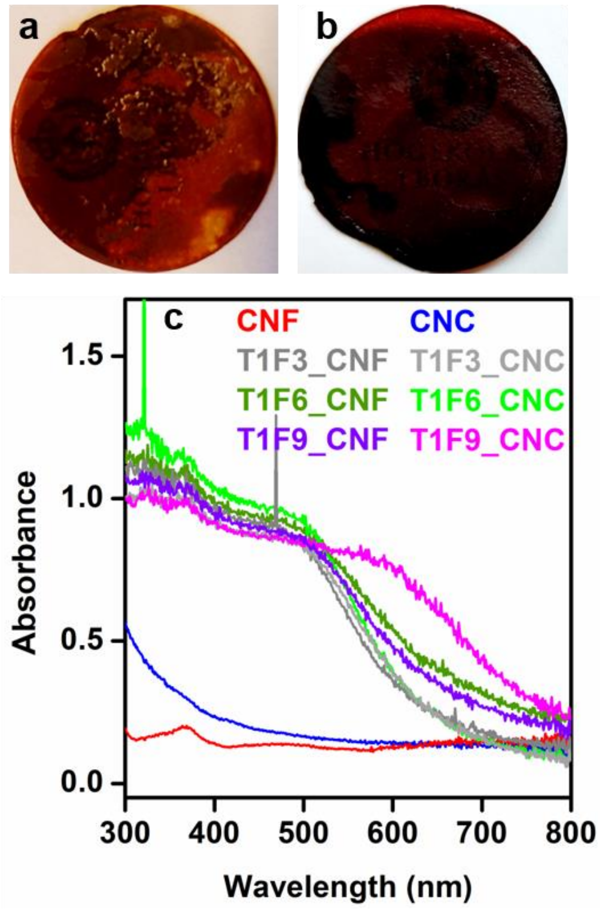

Figure SI9. Photographs of opaque films a) T1F9_CNC, b) T1F9_CNF. c) UV-vis absorption spectra of CNF, CNC and biocomposite films.
